# Supplementary material for: Reversible Ionic Liquid Intercalation for Electrically Controlled Thermal Radiation from Graphene Devices
Source: ACS Nano. 2023 Jun 15;17(12):11583–92. doi: 10.1021/acsnano.3c01698 (PMC7614708; doi:10.1021/acsnano.3c01698)
Supplement: Supplementary file 1 — nn3c01698_si_001.pdf [file nn3c01698_si_001.pdf]

Supporting Information

Reversible Ionic Liquids Intercalation for Electrically Controlled Thermal Radiation from Graphene Devices

Xiaoxiao Yu[1,2], Gokhan Bakan[1,2], Hengyi Guo[1], M. Said Ergoktas[1,2], Pietro Steiner[1,2], Coskun Kocabas* [1,2,3]

1 Department of Materials, The University of Manchester, M13 9PL Manchester, United Kingdom

2 National Graphene Institute, The University of Manchester, M13 9PL Manchester, United Kingdom

3 Henry Royce Institute for Advanced Materials, Royce Hub Building, The University of Manchester, M13 9PL Manchester, United Kingdom

Corresponding Author: coskun.kocabas@manchester.ac.uk

Caption for supporting videos:

Video S1. Real-time recording of the first cycle intercalation with close-up lens of 25μm spatial resolution. The device was applied with bias voltage from 0V to +3.5V by +0.1V increment, followed by deintercalation till -1V.

Video S2. Real-time recording of switching device between high (on) and low (off) thermal emissivity states with mid-wavelength (3-5 μm) infrared camera.

Video S3. Real-time recording of switching device between high (on) and low (off) thermal emissivity states with long-wavelength (8-14 μm) infrared camera.

Figure S1 shows the FTIR results in terms of reflectance of the aluminium foil and polyamide tape. The spectroscopic characterisation uses aluminium foil as the background because of its high reflectivity within infrared wavelength at room temperature (>0.96).[1] Result indicates an average reflectance of approximate 0.9 of the polyamide tape at 7-14 μm wavelength range. Thus, it can be adopted as the other calibration material for thermal characterisation of devices under long-wavelength thermal camera.

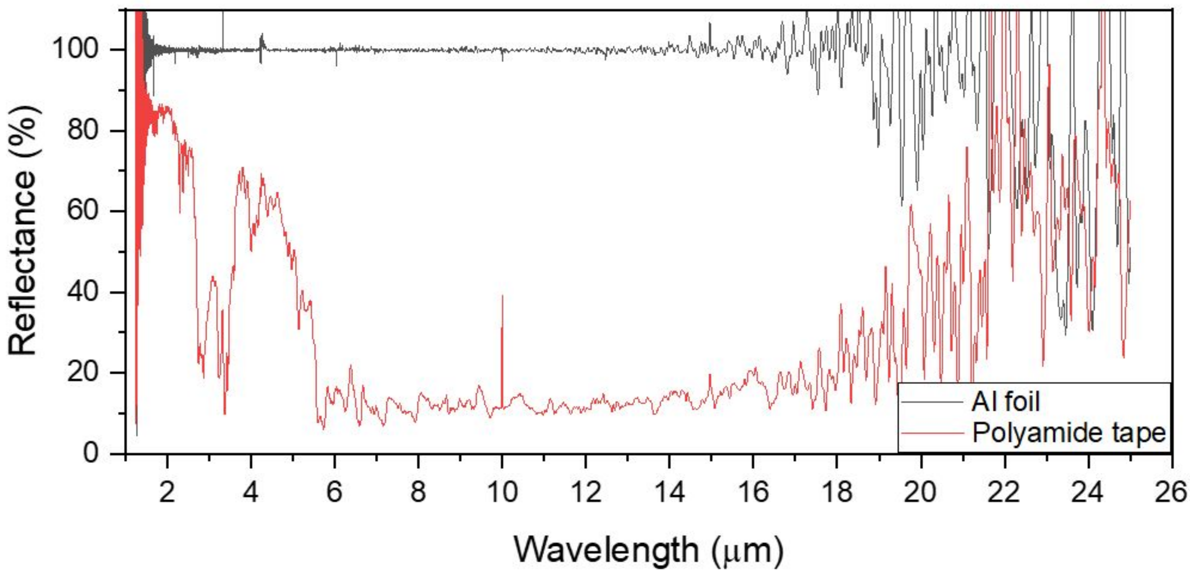

Figure S1. Fourier-transform infrared spectroscopy (FTIR) characterisation of aluminium foil and polyamide tape.

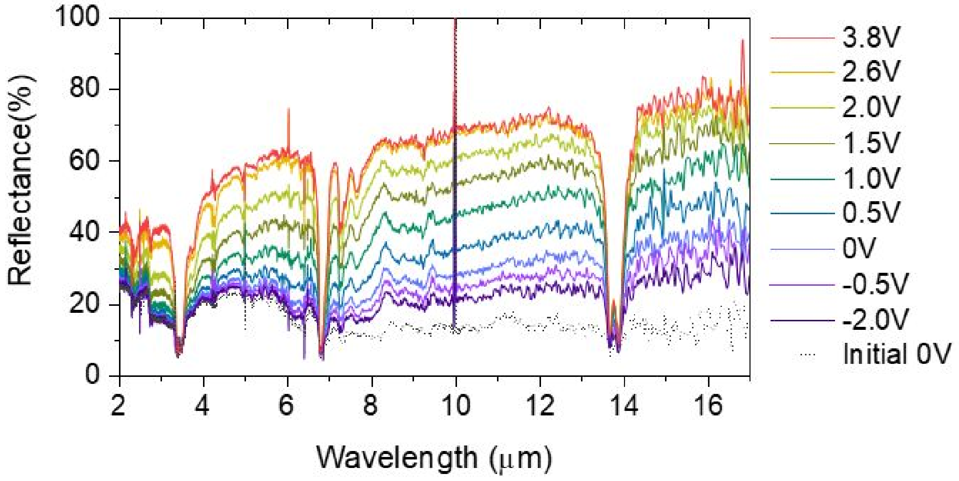

Figure S2. *In situ* FTIR characterisation of a [AMIM][TFSI]-based device at decreasing voltage (from 3.8V to -2.0V).

Figure S2 presents the deintercalation process of the same device in main contents Figure S2a. This FTIR characterisation provides evidence of restoring of the device during the

deintercalation process. It shows that the device is not fully brought back to its initial state at long-wavelength range. This indicates the residue of ions within graphene interlayers or/and structural deformation of MLG. Average results were taken at MWIR (4-5 μm) and LWIR (8-12 μm) to evaluate the thermal modulation performance in both ranges. It shows that the device can maintain at the intercalated state with lower bias voltage (2.6V), and it almost restores to its initial state at small inverse voltage (-2V).

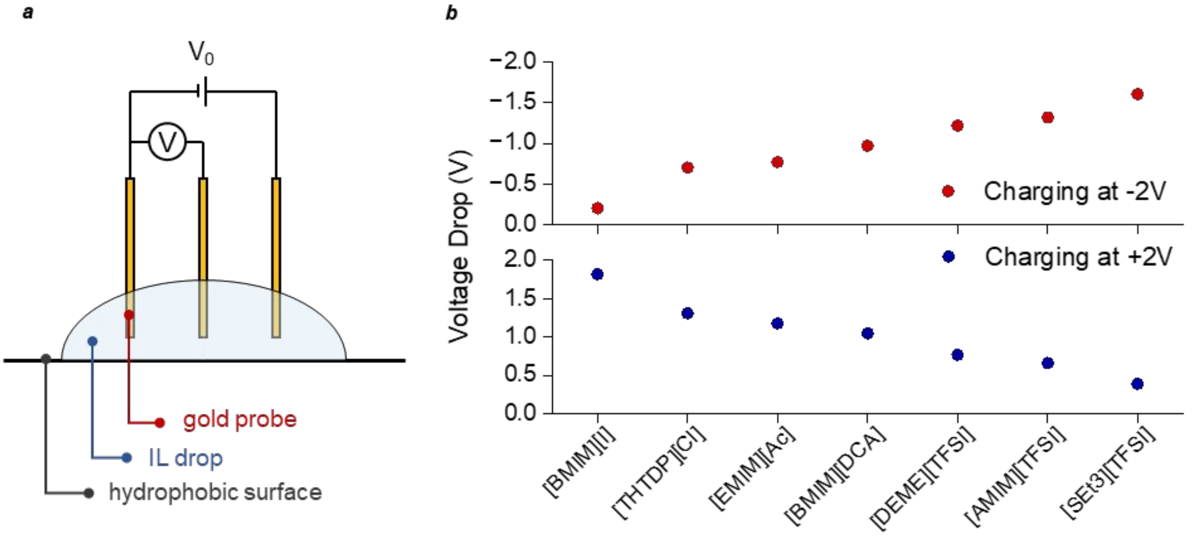

Figure S3. Measurement of asymmetric voltage drop across two EDL. **(a)** Schematic setup of voltage drop measurement. **(b)** Voltage drop across the EDL capacitor formed with different ions of each IL.

The schematic setup in Figure S3a characterises voltage drop of different ions at liquid-electrode interface. Three gold probes were aligned with 2.5mm spacing and immersed into IL drop without touching the non-conductive hydrophobic surface. Overall voltage V_0 was applied with a source meter (Keithley 2400) at +2V and -2V for each IL. The voltage drop V was then measured by the other source meter (Keithley 2400) as the representative of EDL capacitance. Figure S3b verifies that different ion sizes and charge distribution yields asymmetric voltage drop at electrode surface. For instance, both $[I]^-$ and $[SEt3]^+$ are significantly smaller than their pairing ions and present less voltage drop at interface.

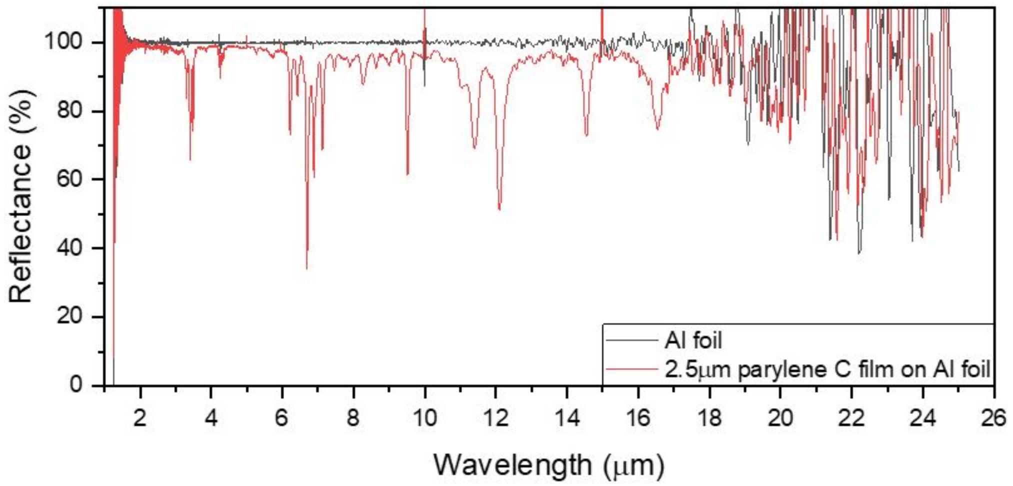

Figure S4. Fourier-transform infrared spectroscopy (FTIR) characterisation of 2.5μm parylene C film on Al foil.

Figure S4 shows that thin parylene C film has small absorption (<10%) within abroad infrared wavelength range except at certain fingerprint absorbing wavelength. Therefore, it can be adopted as the caulk on LDPE film to reduce oxygen content within the device.

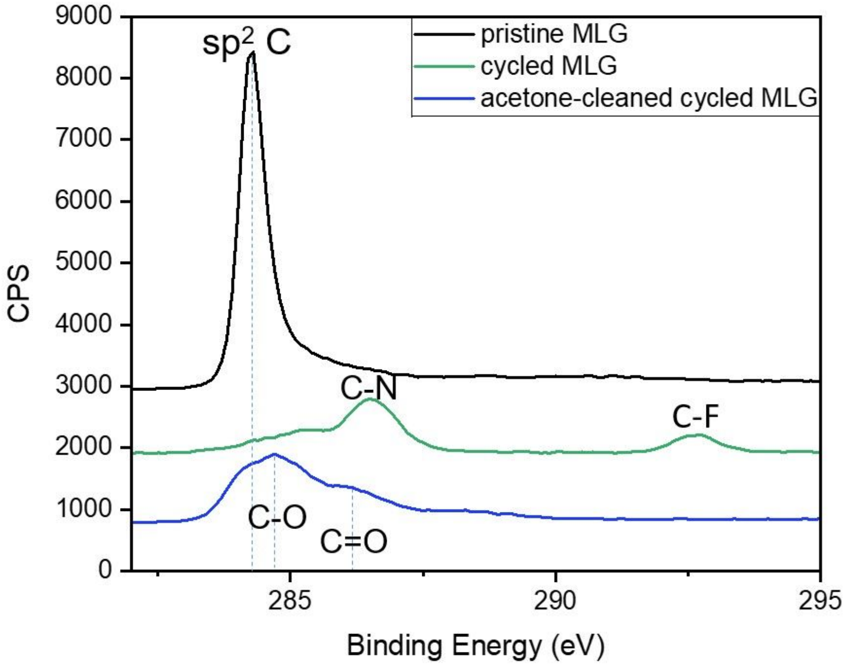

Figure S5. XPS C1s spectra of pristine MLG, cycled MLG, and cycled MLG. After the clycling, we cleaned the MLG with acetone to remove the IL layer. As-grown graphene shows only sp^2-C signal from the graphene layer. After the cycling, C-N and C-F signals appear from ionic liquid. After cleaning the ionic liquid with acetone (C-F and C-N disappear), there is clear C-O and C=O signals indicating oxidation of the graphene layer.

The XPS characterisation was carried out to understand oxidation conditions at MLG surface as shown in Figure S5. The device fabricated with [DEME][TFSI] was cycled with alternative +3.2V and -2V bias voltage until thermal modulation was significantly reduced. The C1s spectrum of cycled MLG denotes the existence of IL at surface. Binding energy of cycled MLG at ~285-286 eV, and 292.6 eV match with the sp^3-C, C-O-N, and CF_3 structure of IL. After gently removing the surface IL with acetone, MLG structure was exposed to X-ray source, indicating that oxidation occurred at its surface.

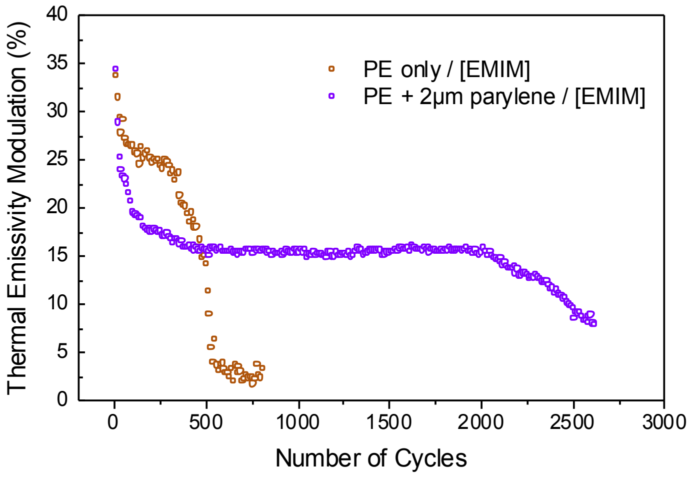

Figure S6. Endurance IR test of cyclic thermal modulation with $[EMIM]^+$ intercalation, based on [EMIM][TFSI] devices with and without parylene coating.

Figure S6 confirms the effect of parylene coating in cyclic performance with device of different electrolyte. It corresponds with previous findings that n-type doping of MLG accelerates the defunctionalisation of the device. Although the parylene coating diminishes oxygen content at MLG surface and extends device lifetime, the electrolyte sacrifices at cyclic intercalation and lowers the thermal modulation depth. Therefore, n-type doped device is not favoured for endured uses in thermal modulation application.

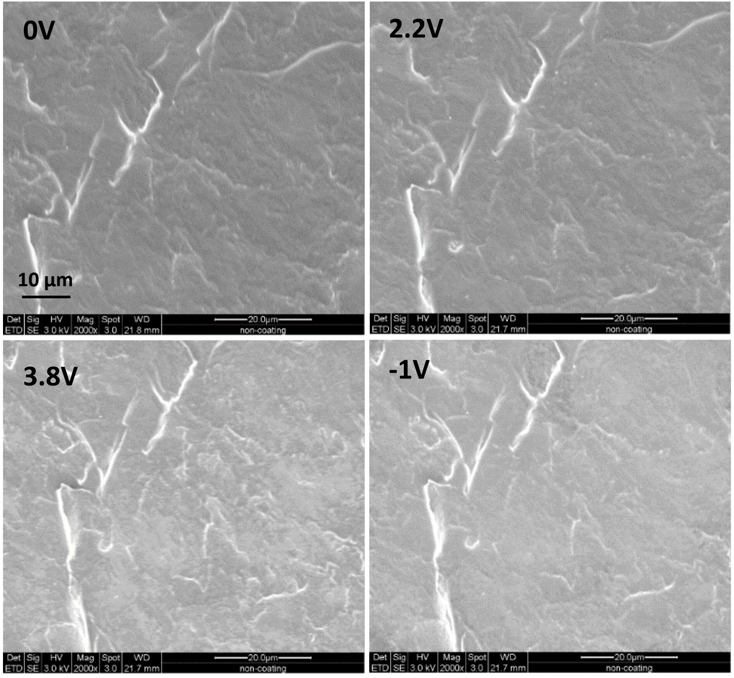

Figure S7. *In situ* scanning electron microscopy (SEM) images of non-coated device surface at initial stage (0V), threshold voltage (2.2V), highly p-doped stage (3.8V), and restored stage (-1V).

Figure S7 shows the *in-situ* SEM characterisation of the intercalation and deintercalation process. It presents a close-up view of surface morphology changes at highly doped stage of 3.8V, and the restoring of the surface structure at inverse voltage (-1V). It indicates that the surface texture is enhanced during intercalation, and the cyclic intercalation process could lead to structural damage in long-term applications.

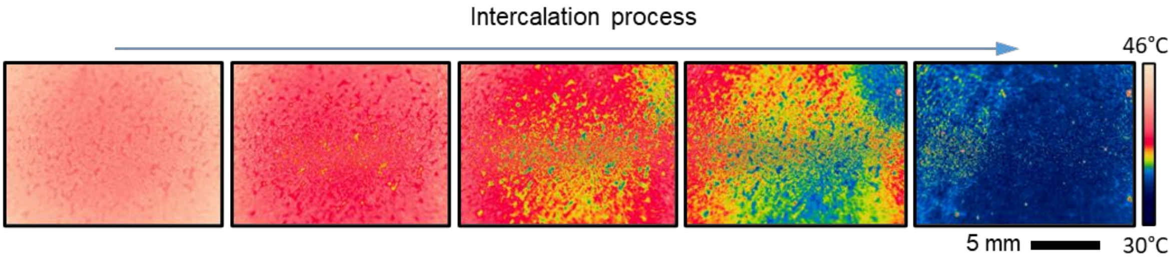

Figure S8. Magnified thermograms showing the intercalation process initiated from the defects on MLG film.

Figure S8 demonstrates the intercalation process with a close-up infrared camera at device surface. It clearly shows that the intercalation process is initiated from the defects on MLG film. Complete recording is available at Video S1.

Reference

1. Bartl, J.; Baranek, M., Emissivity of aluminium and its importance for radiometric measurement. *Measurement science review* **2004,** *4*, 31-36.
